# The Expression of the Claudin Family of Proteins in Colorectal Cancer

**DOI:** 10.3390/biom14030272

**Published:** 2024-02-24

**Authors:** Kristin E. Cox, Shanglei Liu, Robert M. Hoffman, Surinder K. Batra, Punita Dhawan, Michael Bouvet

**Affiliations:** 1 Department of Surgery, University of California San Diego, La Jolla, CA 92037, USA; 2 VA San Diego Healthcare System, La Jolla, CA 92161, USA; 3 AntiCancer, Inc., San Diego, CA 92111, USA; 4 Department of Biochemistry and Molecular Biology, University of Nebraska Medical Center, Omaha, NE 68198, USA; sbatra@unmc.edu (S.K.B.);

**Keywords:** claudin, colorectal cancer, adenocarcinoma, expression, prognostics

## Abstract

Claudins (CLDN1–CLDN24) are a family of tight junction proteins whose dysregulation has been implicated in tumorigeneses of many cancer types. In colorectal cancer (CRC), CLDN1, CLDN2, CLDN4, and CLDN18 have been shown to either be upregulated or aberrantly expressed. In the normal colon, CLDN1 and CLDN3–7 are expressed. Although a few claudins, such as CLDN6 and CLDN7, are expressed in CRC their levels are reduced compared to the normal colon. The present review outlines the expression profiles of claudin proteins in CRC and those that are potential biomarkers for prognostication.

## 1. Introduction

Colorectal cancer (CRC) is the second leading cause of cancer-related deaths in the United States [1]. There are many factors that play a role in the malignant transformation of normal colonic mucosa. A central component of tumorigenesis is the epithelial-to-mesenchymal transition (EMT), which was first described in 1982 by Greenburg et al. regarding its role in embryogenesis [2,3,4,5]. When a cell undergoes EMT, it loses its cell-to-cell adhesion and apical/basal polarity and instead gains the mesenchymal features of motility, invasiveness, and resistance to apoptosis [6]. However, this transition is incomplete, as it is exceedingly rare for carcinoma cells to lose all epithelial markers [7]. Additionally, this process can occur in the reverse, known as mesenchymal to epithelial transition (MET), and is thought to occur at the sites of distant metastases following dissemination [6].

EMT involves the loss of cell-to-cell adhesion and apical/basal polarity. Tight junctions are responsible for these epithelial characteristics. The primary functions of tight junctions are to maintain cell polarity (known as the fence function) and regulate paracellular transport (known as the gate function) [8,9]. Altered or disrupted tight junction proteins have been implicated in tumorigenesis [2,9,10,11].

Claudins and occludins are essential components of tight junctions, which are the most apical connection of epithelial and endothelial cells [2]. In 1998, Furuse et al. first discovered claudin-1 and claudin-2 and named them after the Latin word claudere, meaning “to close” [12]. The claudin family of proteins consists of twenty-four proteins, though the expression of claudin-13 and claudin-24 have yet to be found in human tissues. Claudins contain four transmembrane components, with the N- and C-termini residing within the cytoplasm [9]. Additionally, all human claudins (except claudin-12) contain a motif at the C-terminus that allows for binding to the PDZ (PSD-95/DLG/ZO-1) domains of scaffold proteins [9,13,14,15].

Claudin expression in malignancy is a heterogeneous phenomenon. Downregulation of claudin proteins has been reported in many cancer types, including claudin-2 and claudin-6 in breast cancer and claudin-18 in gastric cancer [16,17]. It is hypothesized that the downregulation of tight junction proteins seen in many cancer types promotes invasiveness, distortion of architecture, and poor differentiation [18]. Despite this apparent advantage of downregulating tight junction proteins, there are a tremendous number of reports on the upregulation of claudins in cancer [10]. This includes claudin-2 in oral squamous cell cancer, claudin-3 in ovarian and laryngeal cancers, and claudin-10 in papillary thyroid cancer [19,20,21,22]. While previous review articles have highlighted numerous claudin proteins’ dysregulation in cancer, there has yet to be a comprehensive report on the expression profiles of the claudin family of proteins in CRC and the ability to use levels of claudin expression for prognostication. The present article will review each claudin subtype and its known expression pattern and role in tumorigenesis.

## 2. Materials and Methods

PubMed was accessed from September 2023 to February 2024. Inclusion criteria included (1) reports of claudin genes in colorectal cancer or the normal colon, (2) non-retracted, and (3) accessible by the University of California, San Diego (UCSD) library. Exclusion criteria included (1) reporting on mouse genes/proteins and (2) expression within inflammatory bowel disease (IBD). For each claudin gene, the phrases “CLD#” OR “claudin #” AND “colon” OR “colorectal” were used as search terms (ex. CLDN2 colon). These criteria returned 562 entries, and each abstract was screened for possible inclusion, after which 245 papers remained and were examined further. Upon reviewing their citations, an additional 38 papers were identified and reviewed. An additional 223 papers were reviewed for background on claudin proteins as well as the expression of claudins within cancers other than colorectal cancer. In total, 172 papers were included in this review article.

## 3. Results

### 3.1. Claudin-1

Claudin-1 (CLDN1), first described by Furuse et al. in 1998, is strongly expressed in the liver and kidney with moderate expression in the lung and skeletal muscle [12]. CLDN1 has been shown to be regulated by the pro-inflammatory cytokine TNF-α and upregulated in areas of active inflammation [23,24]. In oral squamous cell cancer, CLDN1 has been shown to increase cancer cell invasion through the activation of matrix metalloproteinases [25,26].

In the normal colon, multiple groups have used immunohistochemistry (IHC) to determine the expression levels of CLDN1 and found that 76–100% of samples had strong membranous staining (Table 1) [27,28,29,30,31,32]. However, Wang et al. and Gröne et al. reported expression in only 20–25% of normal colon samples, though both had small sample sizes [33,34]. By western blotting, Bürgel et al. reported that 100% of normal colon specimens expressed CLDN1 (*n* = 5) [35]. 

Although there are conflicting reports regarding CLDN1 expression in CRC, the majority of data suggests that CLDN1 is upregulated in CRC. At the RNA level, seventeen independent groups reported the upregulation of CLDN1 in CRC compared to the normal colon [34,36,37,38,39,40,41,42,43,44,45,46,47,48,49,50,51,52,53]. This upregulation was found to occur at all stages of CRC, including metastases [41,42]. It should be noted, however, that most of these studies reported an upregulation based on the average CLDN1 level in CRC compared to the normal colon. When comparing the individual levels of paired samples, however, there is variability. For example, Gröne et al. evaluated thirty paired samples and found that twenty-two CRCs had a statistically significant upregulation, one was downregulated, and the remaining seven were not statistically different from the normal colon [34]. Sewda et al. evaluated over four hundred CRC samples and one hundred normal colon samples, and although the median RNA level of CLDN1 for CRC was greater than the normal colon, almost every value for the normal colon samples fit within the range of the CRC values [46].

At the protein level, CLDN1 was also found to be increased in CRC [34,47,54,55]. Using western blotting, Cherradi et al. found that 92.3% (n = 13) of paired samples showed a significant increase of CLDN1 in CRC compared to the normal colon [31]. Kim et al. also evaluated CRC liver metastases and found that they had the greatest expression of CLDN1, followed by the primary tumor, and then, the normal colon [55]. Shiou et al. reported that 70% (n = 30) of CRCs had high CLDN1 expression while the remainder were found to have low expression [25].

**Figure 1:**
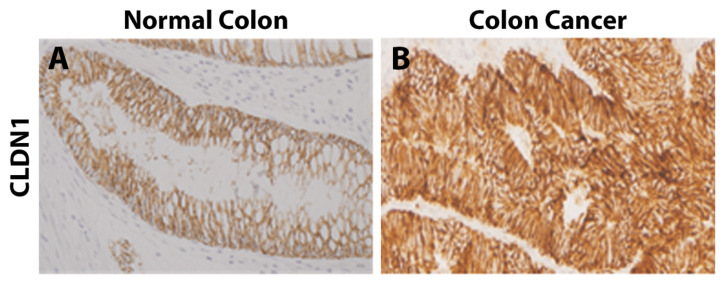
Immunohistochemistry of claudin-1 from Matsuoka et al., *J Surg Oncol* 2011, reprinted with permission [63]. (**A**) Membranous CLDN1 staining of normal colonic epithelium. (**B**) Strong membranous and cytoplasmic CLDN1 staining in colorectal carcinoma sample.

Using IHC, seventeen groups reported that 54–100% of CRCs exhibited CLDN1 staining (Table 2) [27,28,29,30,31,32,33,34,37,46,56,57,58,59,60,61,62]. Multiple groups also directly compared the staining patterns of CRC to the normal colon with two groups reporting that overall, staining patterns were stronger in CRC (Figure 1) [63]. Resnick et al. found that 39.8% (n = 128) of CRCs had increased staining, and Abdelzaher et al. found that 38% (n = 50) of CRC samples had equal staining compared to the normal colon [27,29,44,48].

#### 3.1.1. Changes in the Location of CLDN1 Staining

As a tight junction protein, claudin staining is expected to be confined to cellular membranes. This was found to be true for CLDN1 in the normal colon, with four groups reporting zero cytoplasmic staining, while Bezdekova et al. reported cytoplasmic staining in 5.2% of samples [28,29,30,32,62]. However, in CRC, the incidence of cytoplasmic CLDN1 staining was greatly increased, with reports ranging from 19.4 to 87% [28,29,30,32]. CLDN1 staining was also found to be reduced at the invasive edge of tumors [59,63].

#### 3.1.2. CLDN1 Expression in Colonic Polyps

At the RNA level, CLDN1 was found to be upregulated in adenomas compared to the normal colon (n = 42) [36]. By IHC, CLDN1 expression was found in 51–56% of adenomas [30,31], while Erlenbach-Wünsch et al. reported that 100% of hyperplastic polyps (n = 19) and sessile serrated adenomas (n = 4) exhibited CLDN1 staining [64].

#### 3.1.3. CLDN1 Expression in CRC Metastases

CLDN1 expression was also seen in multiple types of metastases. In the liver, Dhawan et al. reported that 42% of CRC liver metastases had membranous staining while 83% had cytoplasmic staining (n = 12) [32]. Kinugasa et al. found that 92.9% of CRC liver metastases expressed CLDN1, and the only negative sample was from a carcinoid primary tumor (n = 14) [57]. Georges et al. reported that CLDN1 staining patterns in CRC liver metastases were reduced compared to the primary tumor, though 87.5% were still CLDN1 positive (n = 8) [61]. Strong CLDN1 staining was seen in CRC liver metastases (n = 20) compared to normal liver, though when comparing mRNA levels, no significant difference was observed [65]. By western blotting, Kim et al. found that CRC liver metastases had the highest levels of CLDN1, followed by the primary tumor, and then, the normal colon [55]. In CRC lymph node metastases, membranous CLDN1 staining was seen in 31% of cases, while cytoplasmic staining was seen in 38% (n = 13) [32].

#### 3.1.4. CLDN1 Expression in CRC Cell Lines

**Figure 2:**
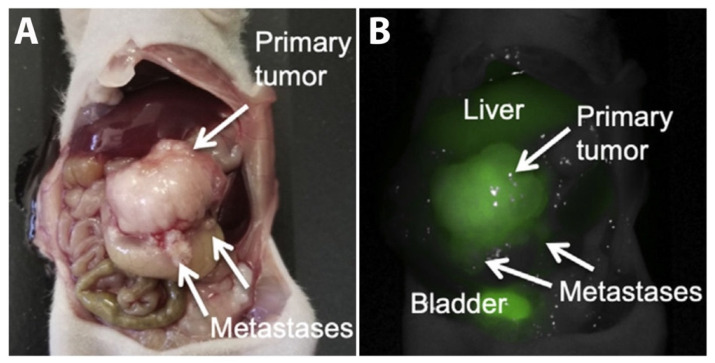
Orthotopic nude mouse model of LS174T labeled with CLDN1-IR800 by Hollandsworth et al., *J Surg Res* 2020, reprinted with permission [68]. (**A**). Bright light image of a large LS174T tumor growing from the cecum with two small metastases. (**B**). Fluorescence imaging of CLDN1-IR800 brightly labeling the tumor and metastases.

By western blotting, the human CRC cell lines Caco2, Colo205, DiFi, HCT115, HT29, KM12, SW480, and SW620 were all found to express CLDN1 [25,31,32,40,41,46,55,66,67]. Those negative for CLDN1 included HCT116, HCT15, and RIE [25,31,32,67]. LS174T and DLD-1 had conflicting reports regarding CLDN1 expression [31,40,41,67,68]. Using an anti-CLDN1 antibody conjugated to a near infrared dye, Hollandsworth et al. were able to brightly label orthotopic CRC tumors grown from the LS174T human CRC cell line in nude mice (Figure 2) [68]. By IHC, Caco2, LoVo, SW480, and SW620 all stained positive for CLDN1 [34]. The overexpression of CLDN1 in both mouse and in vitro studies demonstrated increased tumor growth, development of metastases, and resistance to apoptosis [32].

#### 3.1.5. Prognostication with CLDN1

Multiple groups reported that the loss of CLDN1 in CRC at either the mRNA or the protein level was associated with worse overall survival, while high CLDN1 levels were associated with improved overall survival [29,49,56,58,59,63,69,70]. Zuo et al. performed a meta-analysis and reported that high CLDN1 was associated with a greater overall survival (HR 0.46) [71]. Low CLDN1 staining was also associated with advanced stage, poor differentiation, and positive lymph nodes [58,62,69,72]. However, it was reported that non-responders to the first-line chemotherapeutic agent FOLFOX were more likely to have high CLDN1, while responders were likelier to have low CLDN1 levels [40,41]. 
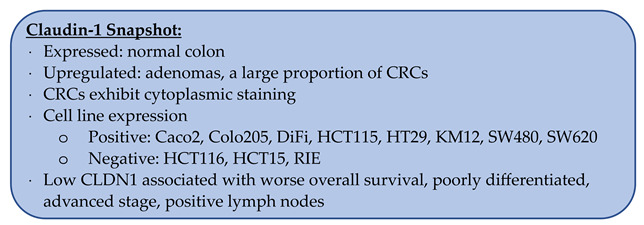


### 3.2. Claudin-2

Claudin-2 (CLDN2), first described by Furuse et al. in 1998, has been found to be highly expressed in the liver and kidney [12,65]. In cancerous tissues, it has been shown to be downregulated in breast carcinoma and upregulated in oral squamous cell cancer compared to normal tissues [16,19].

In the normal colon, there are conflicting reports regarding CLDN2 expression with results ranging from 0 to 100%. Two groups reported complete absence of CLDN2 via western blotting, RT-PCR, or IHC [35,73]. However, Wei at al. reported CLDN2 expression in 10.6% (n = 85), and Dhawan et al. and Hahn-Stromberg et al. reported expression in 100% (n = 13 and n = 32, respectively) of normal colon samples via IHC [74,75,76].

When comparing CLDN2 expression in CRC to the normal colon, there is complete agreement among multiple groups that the average CLDN2 RNA levels are upregulated in CRC [36,39,42,43,48,73,74,75,77]. Similar RNA upregulation was also found to be true in adenomas (n = 42) compared to the normal colon [36]. Additionally, Tabariès et al. reported that CLDN2 expression was more likely to be present in samples with microsatellite instability (n = 377) [78].

**Figure 3:**
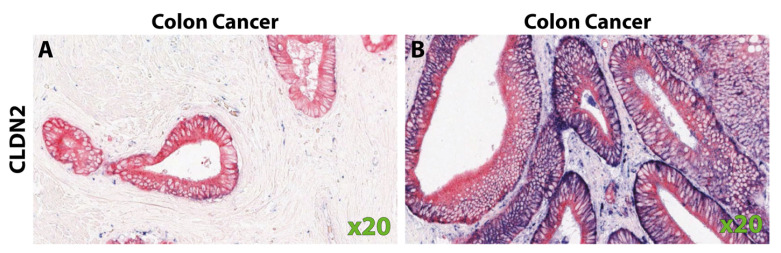
Claudin-2 staining of colorectal cancer specimens from Mezheyeuski et al., *Virchows Arch.* 2018, reprinted with permission [79]. CRC samples without (**A**) and with (**B**) CLDN2 staining.

At the protein level, CLDN2 was found to be upregulated in CRC compared to paired normal samples via western blotting, though sample sizes were small at five to nine in each study [74,75]. By IHC, significant intra- and inter-tumor variability in CLDN2 staining was seen (Figure 3) [79]. The reports on the percentage of CRCs expressing CLDN2 vary from 25 to 100% (Table 3) [73,74,75,80,81]. In adenomas, 27.1% (n = 13) were found to have high CLDN2 expression, while the remaining 76.9% had low expression [75]. When comparing the staining intensity of CRC liver metastases to the primary tumor, 59% (13 of 22) had equal staining, 27.3% (6 of 22) had reduced staining, and 13.6% (3 of 22) had increased CLDN2 staining compared to the primary tumor [78].

#### 3.2.1. CLDN2 Expression in CRC Cell Lines

The CRC cell lines Caco2, DLD-1, HCA-7, HCT15, HT29, LoVo, and SK-CO15 were all reported to express CLDN2 [74,75,78,82]. While HCT116, NCM460, SW403, and SW620 do not express CLDN2 [74,75,78]. SW480 had conflicting reports of absent versus low CLDN2 expression [74,75,82]. Additionally, Ahmad et al. showed that with progressive days in culture, Caco2 no longer expressed CLDN2 while HT29 levels remained stable via western blotting [83]. When CLDN2 expression was induced via a cDNA vector in CLDN2-negative cell lines, they formed more colonies in vitro, and larger tumors grew when implanted into mice [75]. Conversely, when a CLDN2 knockout of HT29 was injected into the spleen of mice, a 2.37-fold reduction in liver metastases was observed [78].

#### 3.2.2. Prognostication with CLDN2

An unpolarized pattern of CLDN2 staining compared to the normal basal pattern was associated with worse overall survival (median 22.8 months vs. 38.4 months), while disease-free survival did not meet statistical significance [79]. High CLDN2 mRNA levels from tumor samples were associated with worse overall survival in three independent datasets as well as a cohort of patient samples [74,82]. Additionally, patients that developed CRC liver metastases within 5 years of diagnosis had increased CLDN2 staining compared to those who did not develop CRC liver metastases [78]. 
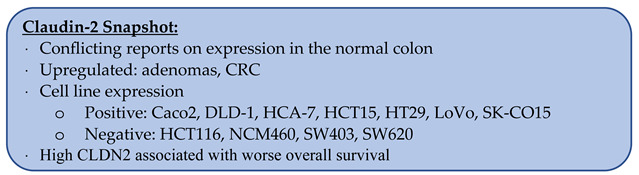


### 3.3. Claudin-3

Claudin-3 (CLDN3) has been found to be upregulated in ovarian, breast, laryngeal, and intestinal-type gastric cancers [20,21,38,84,85,86]. In CRC, there is no clear consensus on CLDN3 expression levels. At the mRNA level, four independent groups were split as to the downregulation versus upregulation of CLDN3 in CRC compared to normal colon samples [43,44,45,87]. Ahmad et al. reported that CLDN3 levels progressively decreased from normal colon to adenoma to successive stages of CRC [88]. However, the range of values for every stage of CRC overlapped with the distribution of the normal colon samples, suggesting high variability among cancer samples.

At the protein level, Bürgel et al. found that CLDN3 was expressed in the normal colon by western blotting (n = 5) [35]. When comparing CRC to the normal colon, three groups showed upregulation of CLDN3 in CRC while Pérez et al. showed downregulation, and Dhawan et al. showed a stable level of CLDN3. It should be noted however that all five studies had modest sample sizes of nine to sixteen [54,75,87,89,90].

By IHC, 58–92.5% of CRC samples stained positive for CLDN3 [91,92,93], and de Mattos et al. reported decreased CLDN3 staining in CRC compared to normal colon samples [94]. There was variability in the reports of CLDN3 expression in the normal colon: Li et al. reported expression in 59% (n = 22), while Ishikawa et al. reported expression in 100% (n = 71) of normal colon samples by IHC [92,93]. There were also conflicting reports on how CRC grade correlated with CLDN3 staining. Li et al. found that CLDN3-positive CRC was more likely to be poorly differentiated, while Ishikawa et al. found that these cancers were more likely to be CLDN3-negative [92,93].

#### 3.3.1. CLDN3 Expression in CRC Cell Lines

The CRC cell lines DLD-1, FET-1, HCA-7, HT29, KM12C, KM12sm, LIM1863, and LS174T were all found to express CLDN3 [66,87,88,95]. Those negative for CLDN3 included DKO-1 and DKS-8 [88]. Caco2, HCT116, SW480, and SW620 all had conflicting reports regarding CLDN3 expression [66,75,88,90,91,95].

#### 3.3.2. Prognostication with CLDN3

Low CLDN3 in CRC at the mRNA level was found to be associated with worse overall survival (n = 250) [88]. 
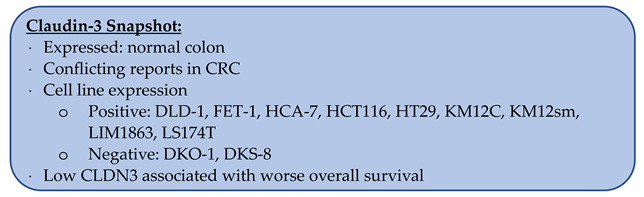


### 3.4. Claudin-4

Claudin-4 (CLDN4) was initially identified as the receptor in which *Clostridium perfringens* interfaces with the epithelial cells of the GI system, creating small pores that disrupt cell permeability and osmosis [96,97]. It has also been shown to be upregulated in ovarian, breast, gastric, cholangiocarcinoma, and pancreatic cancers [20,84,85,86,98,99,100,101,102,103].

In both the normal colon and CRC, there have been conflicting reports on the expression levels of CLDN4. By IHC, four independent groups reported that 100% of normal colon samples expressed CLDN4, though Wang et al. reported expression in only 30% [28,29,33,62,93]. Bürgel et al. found that CLDN4 was expressed in 100% of normal colon samples by western blotting (n = 5) [35].

**Figure 4:**
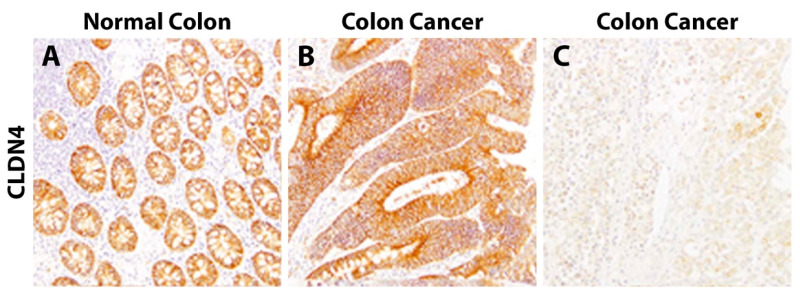
Claudin-4 staining from Fujiwara-Tani et al., *Oncotarget.* 2018, reprinted with permission [104]. (**A**) CLDN4 membranous staining of the normal colon. (**B**) Strong diffuse CLDN4 staining and (**C**) absence of CLDN4 staining in colorectal carcinoma samples.

In CRC, the reports of CLDN4 positivity range from 43 to 100% (Table 4) [28,29,33,59,61,62,93,96]. Resnick et al. reported that 24% of CRC tumors had increased staining compared to the normal colon [29]. Additionally, although Süren et al. found that 87% of the CRCs were found to express CLDN4, all 70 samples had areas within the tumors that lacked CLDN4 staining [62]. Intra- and inter-tumoral variability was also seen by Fujiwara-Tani et al. (Figure 4) [104]. Ueda et al. found that 43% of CRC samples had high CLDN4 staining, and the remaining 57% were categorized as “reduced staining” (n = 129), though no normal colon samples were analyzed for comparison [96].

At the protein level, de Oliveira et al. reported a 2.4-fold increase in CLDN4 in CRC compared to the normal colon [54]. In contrast, Tang et al. found a two-fold decrease of CLDN4 in CRC samples (n = 50) [44]. By RT-PCR, CLDN4 expression was found to be higher in CRC compared to normal colon samples (n = 205) [45].

#### 3.4.1. CLDN4 Expression in CRC Metastases

In CRC metastases, there have been conflicting reports regarding CLDN4 expression. Ueda et al. reported that 68.2% (n = 44) of metastatic lesions had reduced CLDN4 staining compared to the primary tumor [96]. However, Fujiwara-Tani et al. found CLDN4 upregulation in 92.9% of metastatic samples (n = 14) [104]. Holczbauer et al. found strong CLDN4 staining of CRC liver metastases compared to the normal liver (n = 20), though when evaluating mRNA levels, no significant difference was observed [65].

#### 3.4.2. Variation in CLDN4 Staining Patterns

Changes in CLDN4 staining patterns of CRC have also been reported. Matsuoka et al. found that at the invasive margin of tumors, CLDN4 staining was reduced compared to central parts of the tumor [63]. Hahn-Strömberg et al. found that while both normal colon and CRC samples had membranous staining, 25.8% (8 of 31) of CRC samples also had weak-to-moderate cytoplasmic staining [28].

#### 3.4.3. CLDN4 Expression in CRC Cell Lines

The cell lines Caco2, DLD-1, HCT116, HT29, LoVo, SW480, SW620, TCO, and WiDr were all found to express CLDN4 at both the mRNA and protein levels [75,95,96,104].

#### 3.4.4. Prognostication with CLDN4

Matsuoka et al. reported that marked loss of CLDN4 staining was associated with improved disease-free survival compared to mild loss [63]. However, four other groups found that reduced or lack of CLDN4 staining in CRC was associated with higher grade, advanced stage, and positive lymph nodes [62,72,93,96]. 
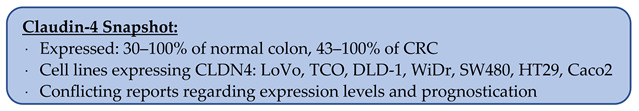


### 3.5. Claudin-5

Claudin-5 (CLDN5) has been found to be expressed in angiosarcomas and benign vascular tumors [80]. Bürgel et al. found that CLDN5 was expressed in the normal colon by western blotting (n = 5) [35]. Reports in CRC, however, are limited, and consist of data only at the RNA level. Using data from both The Cancer Genome Atlas (TCGA) and patient-derived samples, four independent groups reported downregulation of CLDN5 in CRC compared to normal colon samples [36,39,42,43]. Bujko et al. also reported downregulation of CLDN5 in adenoma samples compared to the normal colon [36]. 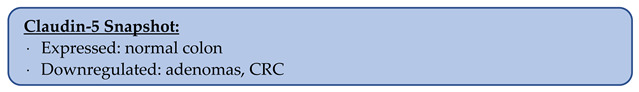


### 3.6. Claudin-6

Claudin-6 (CLDN6) has been shown to be downregulated in breast carcinoma [16]. In CRC, there is no clear consensus regarding CLDN6 expression at either the mRNA or the protein level. Using a TCGA dataset, Alghamdi et al. found that CLDN6 mRNA levels were upregulated in CRC compared to the normal colon [39]. However, Dong et al. reported a reduction in CLDN6 RNA levels in CRC samples. They also compared ten paired samples of CRC and the normal colon by western blotting and found that four had large reductions in CLDN6, while the remaining six had either a minimal change or a slight increase in CLDN6 compared to the normal colon [105].

**Figure 5:**
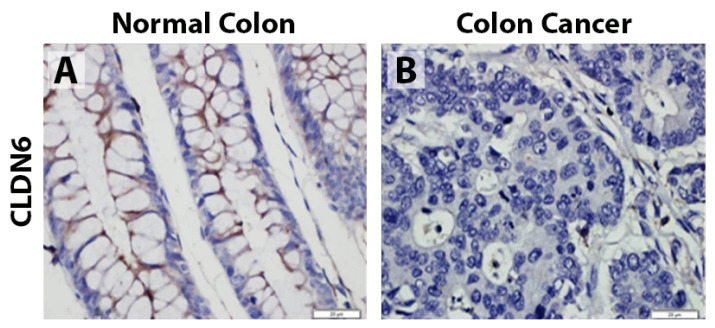
Immunohistochemistry of CLDN6 from Qu et al., *Mol Cell Biochem.* 2022, reprinted with permission [106]. (**A**) Strong membranous CLDN6 staining of normal colonic epithelium. (**B**) Faint CLDN6 staining of CRC. Scale = 20 µm.

By IHC, Qu et al. found that 26.2% (n = 107) of CRCs expressed CLDN6, compared to 75.7% (n = 107) of the adjacent normal colon (Figure 5) [106].

#### 3.6.1. CLDN6 Expression in CRC Cell Lines

By western blotting, HCT116 had low CLDN6 expression, while SW1116 had high CLDN6 expression [105,106].

#### 3.6.2. Prognostication with CLDN6

Qu et al. reported that positive CLDN6 staining was associated with nodal metastases. Of the CRCs that expressed CLDN6, 75% (n = 28) had lymph node metastases, while only 46.8% (n = 79) of those negative for CLDN6 had nodal disease [106]. However, Dong et al. reported that higher levels of CLND6 were associated with improved disease-free survival, with a 5-year survival of ~81% for those with high CLDN6 expression compared to ~60% for those with low levels of expression [105]. 
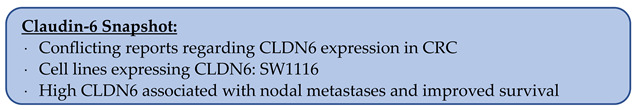


### 3.7. Claudin-7

Claudin-7 (CLDN7) is a unique claudin protein in that it has a strong basolateral membrane distribution unlike other claudins, which are primarily located at the apical surface [107,108,109]. CLDN7 has been shown to be downregulated in lung cancer, and when knocked down in human lung cancer cell lines, cells showed accelerated growth both in vitro and when inoculated into nude mice [110,111]. Downregulation of CLDN7 has been linked to breast cancer as well as invasiveness of both endometrial cancer and esophageal squamous cell carcinoma [38,112,113,114], while the upregulation of CLDN7 has been found in ovarian cancer, chromophobe renal cell carcinoma, and gastric cancer [17,115,116,117,118].

In CRC, data at the mRNA level consistently demonstrate that CLDN7 is downregulated in cancerous tissues compared to the normal colon [39,40,42,43,44,77,119]. Bornholdt et al. also showed this downregulation in colonic tissues as early as mild-to-moderate dysplasia [119]. However, Oshima et al. did not find a statistically significant difference between CRC and normal tissues despite having a sample size of 205 [45].

At the protein level with western blotting, Bornholdt et al. demonstrated a reduction of CLDN7 in CRC compared to paired normal colon samples, though the sample size was only five [119].

**Figure 6:**
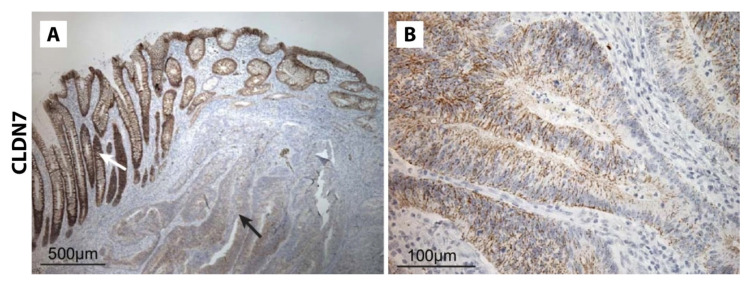
CLDN7 staining of colorectal cancer specimens from Bornholdt et al., *BMC Cancer* 2011, reprinted with permission [119]. (**A**) Reduced CLDN7 staining in area of carcinoma noted by black arrow compared to staining of normal colonic epithelium noted by the white arrow. (**B**) Faint patchy staining of CLDN7 within a carcinoma sample at high magnification.

By IHC, the majority of groups report that the normal colon expresses CLDN7 (Table 5) [28,62,120,121]. In CRC, reports of CLDN7 staining range from 27.3 to 100% [28,62,67,91,109,120,121,122,123,124,125]. However, when comparing the staining of CRC samples to the normal colon, multiple groups have reported decreased expression in CRC (Figure 6) [44,62,119,122,124,125]. This reduced staining was most pronounced at the invasive margin of tumors compared to the tumor core [122,123]. Nakayama et al. reported that 80% (n = 90) of CRC samples had low CLDN7 expression, with 30% or less of the tumor cells positive for CLDN7 [122]. Süren et al. found that 34.3% of CRC samples had loss of staining in more than two thirds of the tumor cells, 42.9% had loss of staining in less than one third of the tumor cells, and 22.8% had staining equal to normal colon tissue [62]. From this, one can reason that at least 65.7% of CRC samples had CLDN7 expression within at least two thirds of the cancer cells.

#### 3.7.1. CLDN7 Serum Levels

Two groups reported reduced serum CLDN7 levels in patients with CRC. Karabulut et al. reported an average serum level in patients with CRC (n = 140) that was 2.3 times lower than healthy control patients (n = 40) (11.6 vs. 26.6 ng/mL) [50]. Xu et al. found that CLDN7 serum levels in patients with CRC (n = 27) were 4.7 times lower than in healthy control patients (n = 9) (15.3 vs. 72.1 pg/mL) [120].

#### 3.7.2. CLDN7 Expression Based on Tumor Differentiation

CLDN7 expression has also been found to correlate with the degree of tumor differentiation (Table 6). Wang et al. found that high expression was seen in 90% of well-differentiated CRCs, 80% of moderately-differentiated CRCs, and 70% of poorly-differentiated CRCs (n = 20 for each group) [124]. Xu et al. found larger differences, with 85% of well-differentiated, 55% of moderately-differentiated, and 28% of poorly-differentiated CRCs exhibiting CLDN7 staining [120].

#### 3.7.3. CLDN7 Expression in CRC Metastases

Holczbauer et al. reported strong CLDN7 staining in CRC liver metastases (n = 20) compared to nearby normal liver [65]. Wang et al. and Kuhn et al. found high CLDN7 expression in 40% (n = 20) and 62.2% (n = 66) of CRC liver metastases, respectively [124,126]. However, Xu et al. reported CLDN7 staining in only 22% [120]. Additionally, 11% (n = 11) of CRC lung metastases and 14% (n = 37) of CRC nodal metastases were found to have CLDN7 expression [120]. Primary CRCs with low CLDN7 mRNA levels were more likely to have liver metastases, 42.2% compared to 22.3% for those with high CLDN7 (n = 102 each) [45].

#### 3.7.4. CLDN7 Expression in CRC Cell Lines

Numerous groups have reported CLDN7 expression via western blotting in CRC cell lines. Those expressing CLDN7 were Caco2, Colo201, DLD-1, HT29, HCT116, LoVo, MDCK, SW480, SW948, and TCO [40,67,75,91,122,124,127,128,129]. Meanwhile, those negative for CLDN7 were Colo320 and YAMC [122,127]. There is conflicting data regarding CLDN7 expression in SW620 [67,91].

#### 3.7.5. Prognostication with CLDN7

Quan et al. found that low CLDN7 expression was associated with worse overall survival and disease-free recurrence [121]. Low or loss of CLDN7 expression was also associated with advanced stage, higher tumor grade, and positive lymph nodes [62,122]. Gowrikumar et al. reported that non-responders (n = 8) to first-line therapy FOLFOX were more likely to have low CLDN7 while responders (n = 8) had high CLDN7 expression [40]. Ianole et al. found that strong CLDN7 staining at the invasive margin, but not at the tumor core, was associated with a worse overall survival [123]. 
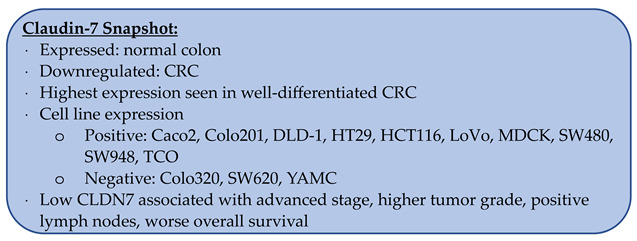


### 3.8. Claudin-8

Claudin-8 (CLDN8) has been implicated in many cancers, including laryngeal, prostate, and osteosarcoma [21,36,130,131]. In CRC, there have been conflicting reports regarding overexpression versus downregulation of CLDN8 compared to paired normal samples. Six independent groups reported that CLDN8 was downregulated in CRC at the RNA level compared to the normal colon [34,36,39,42,43,77]. Bujko et al. also found the same pattern of downregulation in 42 adenoma samples [36]. Gröne et al. showed that in 75% of patients (n = 30), there was at least a 10-fold downregulation of CLDN8. However, no statistically significant difference was seen at the protein level [34]. In contrast, Cheng et al. demonstrated elevated CLDN8 mRNA and protein levels in 20 patient CRC samples compared to normal colonic tissue [132].

#### CLDN8 Expression in CRC Cell Lines

Cheng et al. demonstrated elevated CLDN8 mRNA and protein levels in the CRC cell lines Caco2, HCT116, HT29, SW480, and SW620. With in vitro studies, they found that knockdown of CLDN8 led to reduced cell proliferation, while CLDN8 overexpression led to increased cell migration. Subcutaneous tumor models using the CLDN8 knockdown HT29 and SW480 cell lines showed that the tumors were approximately 50% smaller than those grown from the cell lines with normal CLDN8 expression [132]. 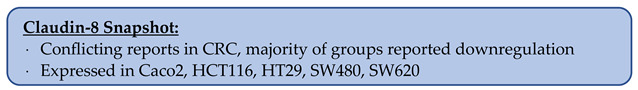


### 3.9. Claudin-9

Claudin-9 (CLDN9) expression within non-neoplastic tissues is limited to the cochlea and the anterior pituitary [133,134,135]. Its upregulation has been reported in diffuse and intestinal-type gastric cancers, endometrial cancer, and hepatocellular carcinoma [118,133,136,137]. In CRC, reports are limited to a single TCGA analysis where CLND9 was found to be upregulated in CRC [39]. 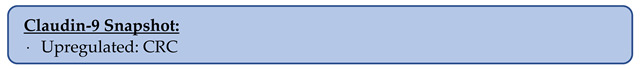


### 3.10. Claudin-10

Claudin-10 (CLDN10) has been reported to be upregulated in papillary thyroid cancer and KRAS mutant non-squamous cell lung cancer and reduced in clear cell renal cell carcinoma [22,138,139,140]. In CRC, a single report by Ahmad et al. indicates weak CLDN10 expression in the CRC cell line Caco2 [88]. 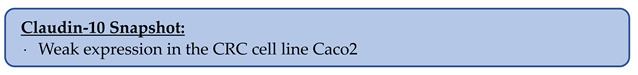


### 3.11. Claudin-11

Claudin-11 (CLDN11) has been reported to be upregulated in breast carcinoma, squamous cell lung cancer, and gastric cancer [16,141,142,143]. In CRC, CLDN11 has been shown to be downregulated at the RNA level [39,43,144].

#### Prognostication with CLDN11

Although CLDN11 RNA levels were generally found to be decreased in CRC, a persistently high level was associated with worse overall survival [43]. Additionally, Li et al. reported that DNA hypermethylation leading to silencing of CLDN11 was associated with metastatic potential and worse progression-free survival [144]. 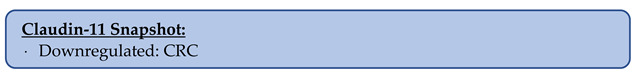


### 3.12. Claudin-12

Claudin-12 (CLDN12) has been shown to be expressed in lung squamous cell cancer and osteosarcoma [145,146]. In CRC, CLDN12 has been shown to be upregulated at the RNA level [39,43]. When directly comparing RNA levels of paired normal colon and CRC samples, Gröne et al. found that 40% of CRCs had a two-fold increase in CLDN12 levels while only 6% showed a significant downregulation (n = 30) [34].

#### CLDN12 Expression in CRC Cell Lines

CLDN12 was found to be expressed in the CRC cell line SW620 [34]. 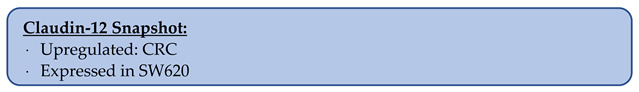


### 3.13. Claudin-13

CLDN13 is not yet known to exist in human tissues [147,148]. 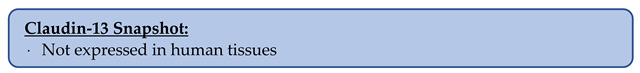


### 3.14. Claudin-14

Claudin-14 (CLDN14) has been shown to be downregulated in breast carcinoma [16]. In CRC, evidence suggests that CLDN14 is upregulated. Multiple groups used TCGA data on colon adenocarcinomas (n = 287) and found that at the RNA level, CLDN14 was upregulated compared to the normal colon [39,43,149].

#### Prognostication with CLDN14

Elevated CLDN14 mRNA levels have been reported to be associated with worse overall survival [43,149]. 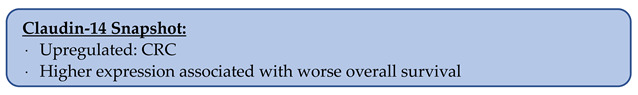


### 3.15. Claudin-15

Claudin-15 (CLDN15) has been reported to be expressed in malignant mesothelioma [150]. In CRC, however, it has been found to be downregulated compared to the normal colon at the RNA level [36,39,43].

#### CLDN15 Expression in CRC Cell Lines

By western blotting, HCT116 was found to express CLDN15 [75]. 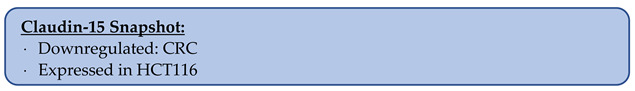


### 3.16. Claudin-16

Claudin-16 (CLDN16) has been shown to be upregulated in breast cancer [151]. In CRC, reports are limited to a single TCGA analysis where CLDN16 was found to be upregulated [39]. 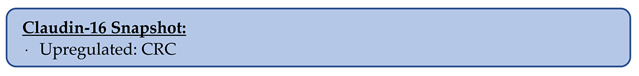


### 3.17. Claudin-18

Claudin-18 (CLDN18) has two alternatively spliced variants, 18.1 and 18.2 [152]. In normal tissues, CLDN18 expression is confined to the lungs for 18.1 and the stomach and duodenum for 18.2 [153,154,155]. In gastric cancers, CLDN18 has been shown to be reduced [17], while its upregulation has been reported in pre-neoplastic conditions such as Barrett’s esophagus, mucinous cystic neoplasms, and intraductal papillary mucinous neoplasms [156,157,158].

The normal colon, however, does not express CLDN18 [159]. When evaluating all subtypes of CRC by IHC, six groups (all with sample sizes greater than 55) reported 1–15% of CRCs expressed CLDN18, while Kim et al. reported expression in 42% of CRCs [152,153,160,161,162,163,164]. Within specific pathologic subtypes of CRC, CLDN18 expression has been reported to be higher: In signet-ring-type CRC, CLDN18 expression was reported at 37.5% while 27.8% of serrated adenocarcinomas expressed CLDN18 (n = 16 and n = 36, respectively) [159,162]. Expression of CLDN18 within the serrated adenocarcinoma subtype was associated with a greater degree of metastatic lymph nodes and advanced overall stage [162]. When evaluating CRC liver metastases, 0% were found to have CLDN18 expression, though the sample size was small at twelve [165].

#### 3.17.1. CLDN18 Expression in Colonic Polyps

There are conflicting reports regarding CLDN18 staining within different polyp types: Sentani et al. reported no expression in either hyperplastic polyps (n = 66) or adenomas (n = 57), while 44.4% of sessile serrated adenomas (n = 45) and 12.8% of traditional serrated adenomas (n = 47) exhibited membranous CLDN18 staining [162]. Kim et al., however, reported higher expression in almost all polyp types. They found that 22.6% of hyperplastic polyps (n = 53), 1.6% of adenomas (n = 63), 35.5% of sessile serrated adenomas (n = 31), and 12.8% of traditional serrated adenomas expressed CLDN18 [164].

#### 3.17.2. Prognostication with CLDN18

CLDN18 staining in CRC was found to be associated with worse overall survival, with a 5-year survival of 0% for those positive for CLDN18 (n = 5) and ~60% for those negative for CLDN18 (n = 92). In multivariate analysis, CLDN18 expression was also found to be an independent predictor of survival [152]. 
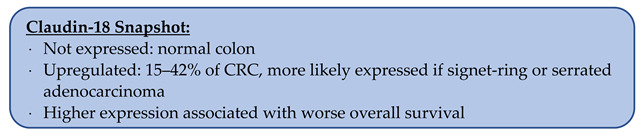


### 3.18. Claudin-19

Claudin-19 (CLDN19) reports in CRC are limited to a single TCGA analysis where it was found to be upregulated in CRC (n = 287) [39]. 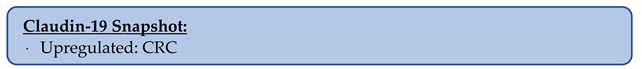


### 3.19. Claudin-23

Claudin-23 (CLDN23), which has been shown to be downregulated in gastric cancer, has also been implicated in CRC [166]. Multiple groups compared mRNA levels of CLDN23 in CRC and found it to be downregulated compared to normal colonic tissues [36,39,42,43,167,168,169]. Bujko et al. evaluated adenoma samples (n = 42) and found the same downregulation of CLDN23 compared to normal colon tissues [36].

#### Prognostication with CLDN23

Yang et al. and Pitule et al. both found that a greater downregulation of CLDN23 was associated with a worse prognosis [43,168]. 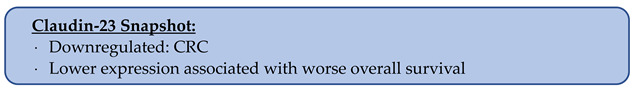


### 3.20. Claudin-24

The Claudin-24 (CLDN24) gene has been found to be located on chromosome 4 in humans, though there are no reports of its expression in human tissues [170]. 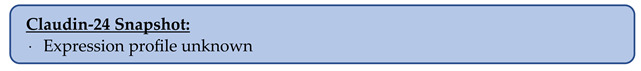


## 4. Discussion

Claudins play a role in normal cellular membrane function, malignant transformation, and tumor invasion. As a class of proteins associated with membrane tight junctions, they facilitate cellular barrier and selective paracellular permeability. In malignancy, however, they have heterogeneous expression profiles and functions. Although often studied in the context of intestinal epithelial cells, claudins also have wide expression in vital organs such as the kidney, skin, and lung [12].

While much work has been performed on this family of proteins, the complete story of their role in cancer remains poorly understood. For example, a single claudin protein (such as CLDN1) may be upregulated in early-stage cancer development only to be reported downregulated in late-stage or more aggressive cancer phenotypes [58,69]. Yet, there are still conflicting reports if some claudins are even expressed in normal colonic tissue, such as CLDN2, or in malignancy, such as CLDN3 [35,73,74,75,76]. There are also discordant findings regarding RNA expression and protein production in some cases, such as CLDN6 [39,105].

Based on the currently available body of work on the protein expression of claudins in colorectal cancer, CLDN1, CLDN2, CLDN4, and CLDN18 have all been reported to be expressed in CRC [27,28,29,30,31,32,33,34,37,46,56,57,58,59,60,61,62,63,73,74,75,80,81,93,96,152,153,160,161,162,163,164]. Although CLDN6 and CLDN7 are expressed in CRC, their levels are reduced compared to the normal colon [44,62,105,106,119,122,124,125]. The remainder of the claudin proteins have yet to be investigated in CRC at the protein level or have conflicting reports at present.

Claudins offer an attractive target for cancer therapeutic and diagnostic strategies due to their transmembrane nature and exposure to the extracellular environment. Despite conflicting reports, dysregulation of claudin proteins is involved in numerous aspects of tumor biology. For example, CLDN1 has recently been proposed as a target for immunofluorescence targeting for improved visualization of colon adenomas and cancers in mouse models [171]. Additionally, CLDN6 and CLDN18 have been proposed as targets for tumor inhibition in proof-of-concept studies [172]. These offer exciting new targets to expand our understanding and management of cancer while calling for highly individualized treatment strategies due to the variability in claudin expression between patients.

## Figures and Tables

**Table 1:** Claudin-1 expression in the normal colon by immunohistochemical staining.

Normal Colon	Normal Cytoplasmic	Study Population	Study
100% (n = 50)	-	Egyptian	Abdelzaher et al. [27]
100% (n = 31)	0%	Swedish	Hahn-Strömberg et al. [28]
100% (n = 129)	-	American	Resnick et al. [29]
94.8% (n = 120)	5.2%	Czech	Bezdekova et al. [30]
87% (n = 45)	-	French	Cherradi et al. [31]
76% (n = 25)	0%	American	Dhawan et al. [32]
25% (n = 16)	-	German	Gröne et al. [34]
20% (n = 20)	-	Chinese	Wang et al. [33]

**Table 2:** Claudin-1 expression in colorectal cancer by immunohistochemistry.

CRC Membranous	CRC Cytoplasmic	Study Population	Study
100% (n = 31)	19.4% (n = 31)	Swedish	Hahn-Strömberg et al. [28]
100% (n = 10)	-	Japanese	Miwa et al. [37]
100% (n = 14)	-	Japanese	Kinugasa et al. [57]
98.4% (n = 128)	-	American	Resnick et al. [29]
96.9% (n = 32)	-	German	Georges et al. [61]
95.7% (n = 23)	-	German	Gröne et al. [34]
91.5% (n = 142)	-	Taiwanese	Dai et al. [60]
87% (n = 120)	87% (n = 120)	Czech	Bezdekova et al. [30]
80% (n = 45)	-	French	Cherradi et al. [31]
76.7% (n = 60)	-	Chinese	Wang et al. [33]
68% (n = 91)	-	American	Sewda et al. [46]
68% (n = 344)	-	Japanese	Shibutani et al. [58]
63% (n = 30)	-	Japanese	Takahashi et al. [59]
60% (n = 50)	-	Egyptian	Abdelzaher et al. [27]
61.4% (n = 70)	-	Turkish	Süren et al. [62]
56% (n = 25)	60% (n = 25)	American	Dhawan et al. [32]
>54% (n = 260)	-	Korean	Kim et al. [56]

Legend: >, data reported as degree of loss of expression (values taken from inverse of samples with “marked loss”).

**Table 3:** Claudin-2 expression in colorectal cancer by immunohistochemistry.

Colorectal Cancer	Study Population	Study
100% (n = 10)	American	Dhawan et al. [75]
100% (n = 33)	Swedish	Hahn-Strömberg [81]
81.8% (n = 11)	Finish	Soini et al. [80]
51% (n = 104)	Chinese	Wei at al. [74]
25.3% (n = 99)	Japanese	Aung et al. [73]

**Table 4:** Claudin-4 expression in colorectal cancer by immunohistochemistry.

Normal Colon	Colorectal Cancer	Study Population	Study
100% (n = 31)	100% (n = 31)	Swedish	Hahn-Strömberg et al. [28]
100%	96.9% (n = 127)	American	Resnick et al. [29]
-	90.3% (n = 31)	German	Georges et al. [61]
100% (n = 70)	at least 87% (n = 70)	Turkish	Süren et al. [62]
30% (n = 20)	85% (n = 60)	not specified	Wang et al. [33]
-	67% (n = 30)	Japanese	Takahashi et al. [59]
100% (n = 71)	59.2% (n = 71)	Japanese	Ishikawa et al. [93]
-	43%	Japanese	Ueda et al. [96]

**Table 5:** Claudin-7 expression in the normal colon and colorectal cancer.

Normal Colon	Colorectal Cancer	Study Population	Study
100% (n = 31)	100% (n = 31)	Swedish	Hahn-Strömberg et al. [28]
-	100% (n = 84)	Romanian	Ianole et al. [123]
32.6% (n = 92)	92.3% (n = 104)	German	Kuhn et al. [126]
-	at least 80% (n = 60)	Chinese	Wang et al. [124]
-	80% (n = 10)	not specified	Hou et al. [125]
100% (n = 72)	74.5% (n = 231)	Chinese	Quan et al. [121]
100% (n = 70)	at least 65.7% (n = 70)	Turkish	Süren et al. [62]
96% (n = 75)	52% (n = 75)	Chinese	Xu et al. [120]
-	50% (n = 100)	Japanese	Tokuhara et al. [91]
-	27.3% (n = 11)	American	Bhat et al. [67]
-	at least 20% (n = 90)	Japanese	Nakayama et al. [122]

**Table 6:** Percentage of colorectal samples with claudin-7 staining changes by degree of differentiation.

Well-Differentiated	Moderately Differentiated	Poorly Differentiated	Study
90%	80%	70%	Wang et al. [124]
85%	55%	28%	Xu et al. [120]
